# Advantage of Medicines in a Liquid Form

**Published:** 1844-04

**Authors:** 


*Advantage of Medicines in a Liquid Form.*—It has been found that fifteen grains of sulphate of quinine, given in infusion of senna, is more efficacious as a tonic, notwithstanding the purgative quality of the mixture, than twenty-four grains of sulphate ef quinine administered in the form of pills. Panizza supposes the causes of this to be that the senna, by promoting the peristaltic action of the alimentary tube, and augmenting the secretion of the bowels, excites the production of a fluid adapted perfectly to dissolve the quinine; and that the quinine, in passing through the intestine in a state of solution, is placed in contact with a much larger extent of surface, and disposed for absorption much more rapidly than if taken in a solid form.—*Panizza, in L'Experience—Lancet,* November 4.

